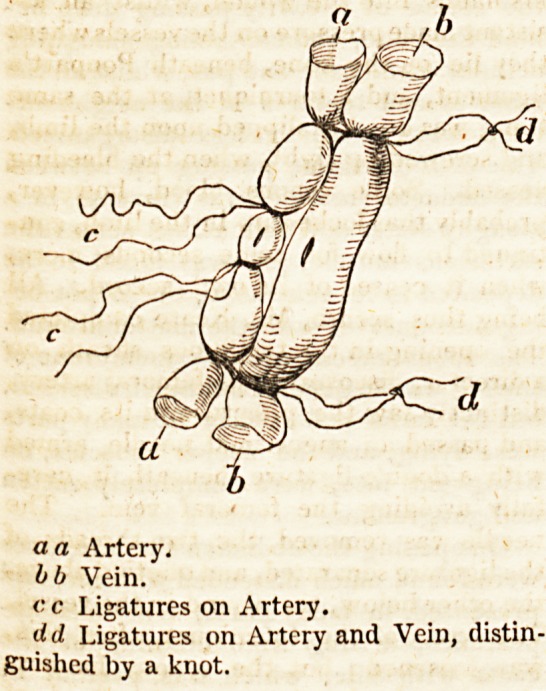# St. George's Hospital

**Published:** 1829-04-01

**Authors:** 


### CLINICAL REVIEW.

### XXXI.

### ST. GEORGE'S HOSPITAL.

LOOSE CARTILAGE IN THE KNEE-JOINT; OPERATION ATTEMPTED WITHOUT SUCCESS.

This case is not devoid of interest, and, therefore, we record it here.

*Case.* Isaac Isbell, ætat. 24, a gentleman's servant, of a strong habit of body, was admitted into St. George's Hospital, September 25th, 1828. In the right knee-joint was a loose cartilage, apparently the size of a small horse-bean, occasioning much inconvenience and rendering him extremely desirous of an operation for his relief. The disease had existed for three years, and latterly had become much worse.

On the 29th, Mr. Brodie, having fixed the cartilage on the inner side of the knee, cut down upon it directly through the skin, when it slipped from under the assistant's finger, and escaped into the joint. No attempts were made to follow it with the knife, and the operation was, therefore, abandoned. Fearing, however, that some coagulated blood had passed into the joint, a cupping-glass was applied to the wound in order to extract it, after which, the edges were united by adhesive plaster, the patella fixed, and the limb placed in a ham-splint. Twenty minims of the liquor opii sedativus were ordered immediately in an ounce and a half of camphor mixture, and cold spirit lotion was applied to the knee. He went on well till the 1st of October, when he suffered more pain in the joint, which continued during the night, and was worse on the 2d, and also accompanied with swelling. The pulse was 90, but moderate in force—the tongue a little white—the bowels confined.

*V.S. ad* ℥xij. *Haust salin.* ℥iss. *Mag. sulph.* ʒj. *Liq. ant. tart.* ♏xx. *6tis horis.*

In the evening, the prescriptions were changed to—*Vin. colchici,* ʒiss. *Haust. salin.* ℥iss. *statim, et repetatur haust. c. vin. col.* ʒss. *6tis horis. Cat. panis. Rep. V.S. ad* ℥xvj.

The pain and the swelling of the joint had at this time rather increased than diminished—the blood was not buffy—the bowels had not acted. He passed a bad night, and the pain and swelling were in much the same state on the 3d, whilst a blush of redness had appeared around the wound, and extended down the leg. The pulse was 96—the tongue a little white, but moist—there had been no rigor—the blood abstracted on the preceding evening presented no buffy coat. *Cont. vin. colch. Omitt. catap. Lot. spt. genu. Empl. lytt. femori.*

On the 4th, there was little alteration in the symptoms or the treatment, but, on the 5th, an abscess had developed itself around the wound, apparently unconnected with the cavity of the joint, and was opened by the lancet. The constitution continued but little affected, the pulse being 90, the tongue clean, the bowels open. A poultice was applied, and, in a day or two, the wound, which had united, and formed a part of the walls of the abscess, gave way to a slight extent, and gave issue to synovia. This, after flowing for several days, ceased of its own accord, the pain and swelling of the articulation rapidly subsided, and, on the 7th, the medicines were finally discontinued. The wound, however, continued very obstinate, and had not entirely cicatrized till the end of November or beginning of December. He was ordered to wear a bandage on the knee, and, being very anxious to return to his situation, was discharged on the 3d of December.

Mr. Brodie, in a valuable clinical lecture on the subject of loose cartilages in the knee-joint, mentioned the particulars of the following interesting case.

A gentleman in the army, who was thought by many eminent surgeons, by Mr. Hey, of Leeds, amongst others, to labour under the affection under consideration, applied to Mr. Brodie, who shared in the general opinion, and advised the application of a bandage. This had been tried before, and the gentleman having made up his mind to undergo an operation, Mr. Brodie consented to perform it. Having kept the patient quiet for two or three days, Mr. Brodie cut down upon what he imagined was the loose cartilage, but which he now discovered was a large fleshy tumour, growing from the inside of the synovial membrane, under the patella. It was now too late to recede, so Mr. B. followed it into the joint, and dissected it out. The patient went on well for twenty-four hours, when the pulse became quick and hard. Mr. B. bled the patient freely—administered a drachm and a half of the vinum colchici forthwith, and half a drachm every six hours afterwards—bled him again, when the pulse got up; and, in short, between the lancet and the colchicum, kept him absolutely in a state of syncope for thirty-six hours. The patient ultimately recovered, but a curious circumstance happened to the joint. The wound had united by the first intention, but, when the inflammation took place in the articulation, and produced a considerable secretion of synovia, the cicatrix first stretched, and at length completely gave way, allowing the collected fluid to escape. The leg was placed in a splint, the synovia continued to run out for a month, when the wound healed, and, as was stated before, the patient, who was an officer in the army, recovered so perfectly as to be enabled to discharge his duties.

Although Mr. Brodie does not consider the operation, under otherwise favourable circumstances, hazardous, yet he has seen it twice prove fatal. In both these cases, the operation was performed under untoward auspices, the first patient having ulceration of the cartilages of the knee-joint, and the second dying of inflammation, ending in suppuration and sloughing under the fascia of the thigh, favoured, Mr. Brodie believes, by a bad state of constitution at the time. On these accounts, Mr. B. recommends the surgeon to take care, before he operates, that his patient is labouring under no other disease besides. To shew how devoid of danger the operation is, when carefully conducted, and in favourable subjects, Mr. Brodie stated that he had taken from the knee-joint of a gentleman five loose cartilages, at three different operations, with success.

### II. Fracture of the Neck of the Thigh-Bone—Delirium Traumaticum—Effects of Opium.

Ann Turton, 70 years of age, was admitted into St. George's on the 23d of January, 1829, under the care of Mr. Cæsar Hawkins, who, we are happy to state, is just elected surgeon to this excellent hospital.

The patient had fallen on her hip, whilst upon the ice the day before, and, since the accident, had been totally incapable of standing, or making any use of the limb. On admission, besides the incapability of moving the limb, and pain on disturbing it in any way, it was found to be shortened three quarters of an inch, and the foot was constantly everted. A crepitus at the hip-joint could be felt, on lengthening and rotating the limb, and the foot could be turned in any direction, though, when left to itself, it always assumed the everted position. The whole thigh was swelled, from extravasation of blood, nearly as low as the knee. She was placed in Earle's bedstead, but could not be made to keep quiet, and, on the 25th, the swelling of the thigh was increased, the integuments were discoloured, and fluctuation was perceived in front. On the 26th, symptoms of the "delirium traumaticum," described by Baron Dupuytren, had shewn themselves, the patient being extremely restless in bed, though still very capable of giving a rational answer to questions. The pulse was weak—the whole surface of the body blue, from imperfect circulation of blood—the thigh less swelled—the hip very tender on pressure, especially in front.

*Eight leeches to the hip—beef tea—arrow-root, &c.—four ounces of wine—forty minims of laudanum directly, and the dose to be repeated, until a decided effect is produced.*

The leeches, by mistake, were not applied, and only sixty minims of laudanum were administered. The delirium became so violent as to need a strait-waistcoat; and, next day, her state was worse than on the 26th. On seeing her, Mr. Hawkins immediately ordered a draught of camphor mixture, tincture of hyosciamus, and carbonate of ammonia, every six hours, with forty minims of laudanum forthwith, and twenty more every second hour. The second dose of laudanum produced complete quiet and relief—the patient obtained much sleep in the course of the afternoon and throughout the night—and, on the 28th, was free from delirium. The pulse was now less frequent—the tongue, which had been deeply furred, was cleaner, though still dry—there was nausea, but no vomiting—less pain in the limb. Half an ounce of castor oil, with twelve drops of laudanum, were directed to be given, and the patient went on well till the evening of the 30th, when, without any obvious cause, the wine and laudanum not having been entirely discontinued, a relapse of the delirium took place, accompanied with such extraordinary depression, that every body thought she was actually dying. At Mr. Hawkins' visit on the 31st, the patient was insensible, and the pulse intermittent. Brandy, wine, beef-tea, &c. were liberally employed, and, next day, (Feb. 1) she had rallied a little, the powers of life not being so low, nor the pulse so intermittent. She dozed much, however, and the tongue was dry and brown. On the 2d, under the continued employment of the same means, the patient had a second time recovered from her delirium, as well as from the dangerous state of depression into which she had fallen. As might, however, be expected, after the administration of such powerful stimuli, some re-action and feverishness now ensued, which was treated by diminishing the quantum of the wine, &c. and exhibiting an ammoniated saline draught, with half a drachm of the tincture of hyosciamus every six hours. On the 6th, the feverishness had subsided, there was no appearance of delirium, and, in fact, whatever the ultimate result of the case may be, the patient had recovered from the effects of her immediate and threatening attack.

This case was exceedingly well treated by Mr. Hawkins, and exhibits the admirable effects of opium in that species of delirium, so near allied in its phenomena to delirium tremens, which follows the reception of severe injuries. Had the patient been treated, as some few years ago she probably would have been, without opium, or even by antiphlogistic means, the fatal results would not have been doubtful. To Baron Dupuytren, the profession are much indebted for calling their attention, in the manner he has done, to the symptoms and methodus medendi of this formidable affection. We would recommend our surgical brethren, in parting, to institute trials, as occasions offer, with opium, administered per anum instead of by the mouth. From our own experience, we are inclined to second warmly this proposal, originating with M. Dupuytren.

---

### XXXII.

ST. GEORGE'S HOSPITAL.

1. CASE OF CUT-THROAT—ULCERATION OF ONE OF THE ARYTÆNOID CARTILAGES—ABSCESS, OBSCURE DURING LIFE, IN FRONT OF THE LARYNX AND TRACHEA.

William Gunyon, ætatis 46, was received into this hospital, on the 27th of January of the present year, having attempted to destroy himself by cutting his throat with a razor, a short time previously. He had lost about a quart of blood, but before his admission some sutures and adhesive straps had been applied, since which but little hæmorrhage externally had taken place. The wound, which was a deep one, was quite to the left of the os hyoides, about an inch below the border of the maxilla, and two inches or thereabouts in length. It was obvious that the carotid artery had escaped, and that neither the larynx nor pharynx were wounded. At the time of his admission he was faint and low, but the pulse soon got up, and he was ordered in the evening thirty drops of laudanum, and twenty minims of antimonial wine in half an oz. of saline draught every six hours. The patient was of a pallid melancholic aspect, was a waiter at a hotel, and for some time past had sunk into a low desponding state of mind, although he had experienced no serious reverses or misfortunes. He had been subject for many years to severe Winter coughs.

On the next day he was going on well, but on the 29th a considerable quantity of coagulated blood being found locked up in the bottom of the wound, the sutures, we believe, were cut away, at any rate a poultice was applied. The blood came away with the latter application, and the wound put on a healthy, though flabby and indolent appearance. On the 1st of February, he complained of much cough, but without any pain in the chest, and half a drachm of paregoric, in an oz. of saline, was exhibited every six hours, with fifteen minims of laudanum at night. He went on well till the 5th, when all at once he was attacked with much pain in the throat, great difficulty of breathing and of deglutition, expectoration of large quantities of thick tenacious mucus, rapid pulse, anxiety of countenance, symptoms in short of a smart attack of laryngitis. About the larynx several enlarged absorbent glands were felt, which contributed no doubt to increase the irritation.

℞ *Hydrarg. submur. gr.* v.—*Op. gr.* iss. *statim. Hirudines,* xij.—*Fotus papaveris. Patient to inhale the steam of warm water through Mudge's inhaler.*

The irritation and inflammation about the larynx were quickly got under by the foregoing remedies, and the patient on the 6th, breathed freely and without pain, though considerable difficulty of swallowing still remained; the pulse was quiet, but as quick as 102. On the 7th, he was given three grains of calomel and a grain of opium at night, and a linctus of oxymel of squills and syrup of poppies was ordered for the cough. On the 9th, he was not so well; the pulse was quick and fuller than it had been, the tongue white and coated, the breathing more difficult, the countenance anxious. There was no attempt at union between the edges of the wound, though its surface was clean, and the skin around was tinted with a slight erythematous blush, which looked suspicious, erysipelas at that time prevailing in the house. The wound was dressed with lint, strips of adhesive plaister, and cold lotion, and salines with antimony again given every six hours. Next day the blush around the wound had disappeared, and the patient on the whole was better, but still the expression was unfavourable, and the cough rather troublesome. Ten leeches were applied to the throat, and followed by eight more on the 11th, when the voice was noticed to be husky and feeble, though the difficulty of breathing and swallowing was diminished. On the 12th, there was little alteration in the symptoms, the cough continuing troublesome, the inspiration being accompanied with rattling in the throat from the quantity of mucus in the trachea, and the sputa of a muco-purulent quality. The pulse expressed, as it had all along, extreme irritability, but no force in the circulating system.

*Omitt. Syrup. et Haust. Salin.* ℞ *Ext. Conii, gr.* iij. *Opii, gr.* ¼. *Cap. quartis horis, velsextis. T. Opii, gtt.* xv. *o. h. s.*

When we saw him on the afternoon of the 13th, he was apparently in a very hopeless situation. The countenance was much depressed—the skin of a somewhat cadaverous hue, approaching in a slight degree the bilious tint observed in those sinking from purulent depôts—the breathing was difficult and painful, especially on coughing, which was frequent—the tongue was dry and glazed, and exhibited a sort of bloodless appearance—the pulse was quick and full, but entirely wanting in force—the whole system in fact was labouring under extreme irritation. It was clear that depletion was out of the question, and one fourth of a grain of opium and one of calomel were ordered every six hours, with a draught of compound spirit of ammonia, and compound spirit of æther, in camphor mixture. Next day he presented no improvement, in fact he appeared to be gradually sinking. A blister was ordered to the throat, but during that night the difficulty of breathing was exasperated, although there was no pain in the neck upon moderate pressure, nor yet in the chest on inspiration. On the 15th, some tumour was perceived for the first time in the lower part of the neck above the sternum, unaccompanied with either pain or tenderness. He was now very anxious, and unable to swallow without coughing—the pulse was extremely rapid, irritable, and weak—the tongue dry and shining, but without fur.

℞ *Mist. Camph.* ℥x. *Tinct. Hyosciam. m.*xxv. *Spirit. æth. c. m.* xx. ♏ *6tis horis.*

Mr. Hawkins could not satisfy himself of the presence of fluctuation in the swelling above described, nor could any body else, and therefore, as its nature was dubious and the patient sinking, no incision was performed. In the afternoon of the 16th, he died, the tumefaction having previously appeared to get something less.

*Sectio Cadaveris.* The wound although gaping, was otherwise healthy and had no sinus leading to or from it. In the front of the larynx and trachea was a large collection of pus, extending from the os hyoides to beneath the sternum, when the deep cervical fascia prevented its further descent into the chest. Laterally it passed under the sterno-mastoid muscles, and more especially at their lower part. In front it had dissected from the larynx and trachea, the sterno-hyoid and thyroid muscles, beneath which it lay, and by which it had been prevented from pointing at the surface. The abscess had no distinct cyst, but had burrowed in the cellular membrane beneath and between the muscles above-mentioned. The abscess had no communication, or none that could be found, with the cavity of the wind-pipe, it certainly had none with the wound. On opening the larynx, the membrane passing from the epiglottis to the arytænoid cartilages was found to be a good deal thickened, and presented at one part a small and circular depression, apparently from ulceration. On the right side of this a little sinus led into a cavity, no doubt that of an abscess, surrounding the right arytænoid cartilage, which was partly ossified, almost entirely denuded, and portions of it lay loose in the cavity described. The cavity had no communication with the external wound, though only a membranous septum intervened; the arytæno-cricoid joint was sound. The mucous membrane of the trachea and bronchi was very much inflamed—both lungs contained a quantity of muco-serolent fluid—the lower lobe of the right lung was condensed—and, the pleura investing it was coated with patches of recent lymph. The head was not examined.

On reading the dissection of the foregoing case the question, we think, must occur to all, "what gave rise to the abscess in front of the larynx and trachea?" It was not matter burrowing from the wound, for with that it had no connexion,—it was not the direct extension of the inflammation from within the trachea and larynx to without, for with their interior it had also no communication nor connexion. If it be said, "then on what did it depend?" we answer, that we really do not know. Mr. Hawkins observed in the dead house, that it probably was the consequence of the ulceration of the internal membrane of the larynx, and stated that he had witnessed a case or cases of such inflammation or ulceration, accompanied, as here, by external suppuration. We cannot help suspecting from the insidious manner in which it formed, as well as by the symptoms of extreme irritability and depression with which it was accompanied, that the cause of the abscess was something more than this. However, conjectures are unprofitable things at the best, and we gladly waive them altogether.

### II. REMOVAL OF PART OF A THICKENED PREPUCE.—INSIDIOUS PLEURITIS AND PNEUMONIA.—DEATH.

A young man of four or five and twenty, applied at the Hospital on Mr. Keate's admission day, Feb. 18th, of the present year, with the prepuce much thickened after paraphymosis, and a part of it attached to the frænum, forming a kind of tumour, the size of a cherry, beneath the glans. He was taken into the house, some physic was given him, and the tumour, if such it be called, removed on the 20th, by Mr. Keate, certainly no very formidable operation. On the next day, the prepuce was a good deal swollen, and a good deal of feverishness was present, for which the patient had salines with the sulphate of magnesia and antimonial wine every six hours. The part itself was fomented, and on the 22d, there was nothing particular observable about him or it, save that he was, as indeed he had been all along, unusually nervous and irritable. Some more of the common black draught was prescribed, and at the house-surgeon's visit on the 23d, the penis was still rather swelled, but the man's condition otherwise excited most surprise. His countenance was sunken, pale, and anxious—the breathing was hurried—the pulse quick and small—the skin hot—the tongue white and furred—the bowels still confined. He had no cough whatever, and could take in a full inspiration with ease. Calomel, haustus sennæ, and a repetition of the salines were prescribed, but in the evening he was still very irritable and low, the pulse was without any force, the countenance much depressed. Twenty minims of laudanum with syrup in camphor mixture were prescribed by the house-surgeon, but at eight o'clock next morning he was evidently sinking. The spirits of sulphuric æther were added to the salines, and a blister to the side of the chest was in contemplation, but the patient soon saved those concerned any further trouble, for he died at 12 o'clock.

*Sectio Cadaveris.* The face had a slight tinge of yellow, at any rate, it was certainly more sallow than usual. On raising the sternum, the veins passing to the right side of the heart were gorged with black blood. The pleuræ, pulmonalis and costalis, of the right side were extensively united by old adhesions, intermingled with some fresh ones. The middle and inferior lobes of the right lung were bound together by a flake of strong, recent, and yellow lymph, which appeared half verging into pus, whilst the lung itself presented strong evidence of acute inflammation of its parenchyma, being generally full of blood, and in some places, almost hepatized. The pleuræ on the left side were also united by old adhesions, intermixed with a few flocculi of recent lymph. The lung on this side was very extensively affected with hepatization, the effect of recent pneumonia, though a few, and but a few, air bubbles could be squeezed from the parenchyma. The right chambers of the heart were gorged with black blood, and the left were more filled with the same than they usually are. On removing the calvarium the veins of the dura and pia mater were turgid, and the arachnoid was opaque for some extent of surface, evidently not from recent inflammation. The brain was very firm, the black points in its substance numerous, a little serum was contained within its ventricles. The liver and the other abdominal viscera were healthy.

Nothing particular was observed about the penis or the wound, which appeared to be nearly healed. There was no suppuration, or any thing like it, and little or no tumefaction of the prepuce.

The case, though not so rare as to become a matter of wonderment or mystery, is yet not so common as to merit being passed entirely sub silentio. The pleuritis and pneumonia were evidently called into life by the operation, slight and unimportant as that would seem to be, and are calculated to show how dangerous in certain constitutions, and especially, it would appear, in hospital practice, very trifling wounds will occasionally prove. By the bye, this case and others of the same description, are sore stumbling blocks to those who see nothing but vitiation of the blood, and make the depôts of pus in the lungs or the liver, to be owing to a kind of *transubstantiation* of that which exists in the wound. The pleurisy or peritonitis which follow hard upon an injury, and the abscesses in the liver or the lungs which occur at the end of a certain number of days, are evidently only varieties of the same constitutional implication, and yet the former are a monstrous deal more difficult to explain on the humoral pathology than the latter. Would Mr. Arnott argue that the pneumonia in the present instance depended on inflammation of the veins of the penis?

### III. RANULA—TREATMENT BY THE SETON.

*Case.* Sarah Rivers, a stout but rather strumous looking country girl, ætatis 23, found a tumour appear beneath her tongue, without any known cause, and gradually increase, unaccompanied by pain. On the 4th of last December, five months after the commencement of the disease, she entered the hospital, under the care of Mr. Brodie. The ranula, for a ranula it was, occupied its usual situation beneath the tongue, was the size of an almond, of oblong form, soft and fluctuating, its surface of natural colour, at one part slightly transparent. It gave no pain, but much inconvenience in talking.

On the 8th, Mr. Brodie passed a seton through the tumour, when ʒj. of glairy fluid escaped. On the 13th, she was feverish, and was ordered some calomel and antimony, with senna, and, on the 15th, complaining of pain in the forehead, leeches were applied to the part. After some little time the seton was drawn out, and the girl seemed cured; but the fluid again collected in the tumour, and another seton was introduced on the 20th. On the 27th, the swelling was greatly reduced again in size, and the girl was sent into the country, with directions to allow the seton to remain, until it should come out by ulceration.

*Case* 2. A woman apparently some six or eight and twenty years of age, presented herself at Mr. Brodie's visit to the hospital, on the 23d February, 1829, with a large ranula. The tumour was the size of a small egg, and accurately filled up the space between the teeth of the under jaw and beneath the tongue. Fluctuation was distinct, and the mucous membrane, the distension of which occasioned the tumour, was translucent in parts, and no where thickened. It had commenced, the patient said, three or four months before the present time, and prevented her articulating certain words distinctly; but, on asking her to speak, the voice had certainly none of that croak the name of the disease is meant to imply.

Mr. Brodie having armed a curved needle with a thick, double ligature, passed it through the ranula transversely, cut away the needle, and left the ligature behind, to serve the purpose of a seton. Mr. Brodie then punctured the anterior part of the tumour with a lancet, and let out some two or three drachms or more of a thick, gelatinous, transparent liquid, which coagulated a good deal on heat, but did not become opaque. The ends of the seton were loosely tied together, to prevent it slipping out, and the noose bound down to the chin by a strip of plaister. The woman then went home, with directions to show herself occasionally, but on no account to take the seton away, until it should separate itself by ulceration.

We detail these two cases, as shewing the practice of Mr. Brodie, in this trifling, perhaps, but still very troublesome affection. The plan recommended in many, if not most, of our systematic books upon surgery, viz. puncturing the tumour, and allowing its contents to run out, is really not worth a farthing in practice, for the ranula almost invariably returns. Even the more powerful application of the seton is not always successful; far from it; but still Mr. Brodie has found it answer better than any other means he has employed. The issue of the first case shews that it is necessary to keep the seton in until it is discharged by ulceration, in order to ensure any sort of success. We make no apology for introducing what may seem to be a minor description of cases in surgery, because we conceive it requires none. If pupils had not such an unconquerable mania for running only after "great operations," and the public for relishing nothing but accounts of "great cases," it would certainly be better for both. The sentiments of Mr. Pott on this subject, in the preface to his paper on the fistula lachrymalis, deserve to be written in letters of gold. "The operative part of surgery," says Mr. Pott, "is far from being the whole of it; and I cannot help thinking that, by attending a little more to what is called common or practical surgery, our art might still be considerably improved, practitioners rendered more expert, and mankind much benefited."

### IV. Scalp Wound—Erysipelas of the Head and Face.

Saint George's Hospital has long been noted for the prevalence and frequent severity of erysipelas within its walls. Within the last one or two years, however, a comparative immunity from the assaults of St. Anthony had been enjoyed, till the latter end of the present, or rather the past, Winter, when it again became almost epidemic, and in three or four instances proved fatal. It first appeared about the breaking up of the first frost, and from that until the present time of writing, March 1st, it has continued more or less to prevail. Mr. Brodie has observed that, consideratis considerandis, erysipelas has been more extensive, and its characters more severe during or after a long continuance of easterly winds, than at any other time. In January and February of the present year, we have notoriously had a great deal of those particular winds, and not only that, but sudden and almost unusual variations of temperature, circumstances which conjoined are especially favourable to the generation and extension of erysipelas. The following case we select from many we have witnessed, as it was rather a severe attack, and exemplifies well, or at least as well as a single case can do so, the principles of treatment pursued in the surgical wards of this hospital, which has, somewhat uncourteously, been termed "a hot-bed of the disease."

*Case.* William Kelly, a healthy-looking young man, received, on the 17th of February last, a trifling scalp-wound on the posterior part of the side of the head, by which the pericranium was denuded, but not the bone. He came to the hospital and was seen by Mr. Harrison, the house-surgeon, who, finding no symptoms of concussion or compression, dressed the wound lightly, gave him some infusion of senna, and sent him home with directions to keep quiet. On the 19th, he returned with some sharp febrile symptoms, and looking very ill. Mr. Harrison pressed him to enter the hospital, but having some family affairs to settle, he could not come in till the next day, the 20th, when he fell to the care of Mr. Keate.

At this time he complained of much pain in the head, had experienced some rigors, the pulse was full and hard, the tongue white and coated. There was no appearance of redness, tumefaction, or erysipelas about the scalp, but the lips of the wound were asunder. He was bled to eight or ten ounces, and ordered five grains of calomel immediately, and the common house-physic in three hours afterwards. Salines with a drachm of the sulphate of magnesia, and fifteen minims of antimonial wine were directed to be taken every six hours. In the evening the bleeding was repeated, and the blood drawn on both occasions was highly buffed and cupped. On the 21st erysipelas appeared upon the scalp, the pulse was 112, the skin hot, the bowels freely open, the tongue white and furred.

℞. *Liq. ammon. acet.* ℥ss. *aq. distil.* ℥j. 6*tis horis.*

The erysipelas extended over the forehead, accompanied with an œdematous condition of the scalp, particularly at or near the injured spot, and a slight degree of puffiness in the immediate vicinity of the wound. The treatment was continued throughout the 22d, without alteration, but on the 23d, the pulse being quicker and not so full, the skin pretty cool, the tongue moist and coated, the bowels rather purged, and the erysipelas spreading on the face, the medicine was changed to half an ounce of the liq. ammon. acet, and ten minims of laudanum, in an ounce of camphor mixture. On the 24th, the pulse was quick, but devoid of any thing like force; the face was generally swollen, and of dull red colour, the tongue was getting brown in the centre, and coated white at the edges, the mind was inclined to be rambling and incoherent, though the patient answered questions very properly. Bark was now given in the form of an ounce of the decoction, and half a drachm of tincture, with the liquor ammoniæ acetatis as before, every six hours.

On the 25th, the erysipelas was scaling on the forehead and face, but still the swelling continued undiminished or nearly undiminished, the brownish red colour of the parts remained, the patient was more light-headed, the pulse was lower than it had been yet, the tongue more brown and dry. Since the first few days he had had no rigor, and the puffiness and œdema about the head were less; the bowels had not been opened since the evening of the 24th.

*Repetatur decoct. cinch. addendo tinct. ejusdem,* ʒss.

26th. No worse, which in these cases is generally considered as better. The pulse has nothing unfavourable about it —the tongue is dry and rough, and coated in the middle with a thick brown fur down to the apex—the face much swollen but scaling—the scalp and the wound much the same. He does not, and has not for several days, complained of any head-ache; has had no rigor nor vomiting, and answers questions rationally enough. The parts had been hitherto treated with cold lotions, but to-day a linen mask smeared with erysipelas ointment is substituted for them. He is obliged to be confined in a strait-waistcoat, not on account of being furious or delirious, but in order to prevent his pulling the rags off his face, which he constantly does. The bowels have not yet been opened. Mr. Harrison ordered some house-medicine, which soon produced an evacuation from the bowels, and on the 27th, the patient was remarkably improved. The tongue had become moist, and was much cleaner—the pulse was quiet—the skin cool—the erysipelas scaling. The patient gradually convalesced under the employment of bark, and afterwards gently nutritious diet, and had no further unfavourable symptom.

Here we have a single case of erysipelas treated by bleeding, salines, and bark, according to its varying features. With respect to the first-mentioned remedy, bleeding, we may state that it is seldom or never employed at this hospital; indeed the only instance we remember, besides the present, was one of erysipelas complicated with injury of the head, when the lancet was resorted to, and that to a very considerable extent, with the very best effects. The common run of cases are treated by calomel and antimony, salines with antimony, &c. holding back the bark till the febrile symptoms have passed, or are passing, and the tongue is cleaning. There is, however, a class of cases, especially of erysipelas about the head and face, where to wait for the cleaning of the tongue, before the exhibition of bark, would probably be to wait for what never would arrive, at least on this side the dead-house. Such a case (but not a severe one) was the preceding, and the good effects of the cinchona, when the pulse got quick and low, and the tongue was growing brown, will be evident, we conceive, to all who peruse the details we have given. We shall return to the subject as opportunities offer, in order to shew the general practice in erysipelas pursued at this hospital, both as regards the "internal" remedies employed, and the local.

### LXIV.

### ST. GEORGE'S HOSPITAL.

### WOUND OF THE FEMORAL ARTERY AND VEIN IN THE SHEATH OF THE TRICEPS —GANGRENE OF THE LIMB—DEATH.

The case we shall detail occurred very lately at St. George's Hospital, excited much interest, and is highly deserving of attention.

A man of the name of Timms, 32 years of age, was cutting out a little boat for his child, when the knife, a common clasp one, similar to that which is commonly used by labouring people, slipped from the wood, and the point stuck into the left thigh. An immediate and profuse hæmorrhage from the wound was the consequence, and he swooned, or nearly swooned, before any one could come to his assistance. His master, a carpenter or dealer in timber, immediately applied a fillet of coarse list round the limb, above the wound, for the purpose, as he said, of more effectually stopping the bleeding, and Mr. Wilson, a surgeon, residing at Fulham, where the accident happened, was summoned to attend. With the assistance of Mr. Wilson and his master, the man was conveyed to St. George's Hospital in a coach, and admitted at 5, p.m. During the time he remained in the coach, no bleeding would seem to have occurred; but, owing to the slipping of the fillet, or some accidental circumstance, it burst out afresh at the hospital door, and the passage from that to the bed upon which he was laid in the ward, presented a kind of rivulet of blood. Compression on the vessels at the groin was immediately made by the house-surgeon, Mr. Harrison, when the hæmorrhage ceased. The compression was continued by means of the key-press, and, ten minutes afterwards, when we saw him first, we found the patient in the following condition.

He was lying extended on his back, in a state very nearly approaching to syncope, with the face and surface of the body blanched, the skin cold, and the pulse at the wrist very feeble and tremulous. Besides these symptoms, the patient had the yawning and feeling of sickness dependent on great loss of blood. Two-thirds down the thigh, on its inner side, just in the situation of the femoral artery, where lodged in the sheath of the triceps adductor, was a transverse wound, scarce an inch in length, which appeared, from its position and direction, to have passed through, or near to, the sartorius muscle. The limb was by no means swelled, nor had any material degree of extravasation taken place into its textures, immediately surrounding the puncture. As the hæmorrhage was commanded so completely by the pressure at the groin, it was thought by all present that no sort of necessity existed for the tourniquet, which would tend to produce much swelling in the limb, and obscure the steps of that operation which the surgeon would probably perform.

Summonses were immediately issued to Mr. Keate, the surgeon of the week, and Mr. Hawkins, and every thing, in the interim, was prepared for operation. Whilst Mr. Good, who before was making pressure, and the house-surgeon, went out of the ward for some necessary purpose, the compression at the groin was entrusted to Mr. Wilson. The pad, however, slipped in some way or other, and the house-surgeon was summoned again in a hurry, on account of a return of the bleeding. He instantly ran to the spot, and found that blood was trickling down the thigh, and a good deal effused about the wound in the cellular texture. The key-press was re-applied, and the hæmorrhage ceased.

At 6, p.m. Mr. Hawkins, and soon afterwards Mr. Keate, arrived, when the latter, on ascertaining the nature of the case, immediately determined to secure the vessel, above and below the puncture in its coats.

An incision, some three or four inches in length, following the direction of the femoral artery, and having the original wound in its centre, was made through the integuments and cellular membrane. Some firm coagulum was turned out, and the musculus sartorius exposed. This had been wounded at its lower or outer edge, if not transfixed in the centre by the knife, but the wound being, at all events, *towards* its inferior margin, the muscle was turned up, and Mr. Keate continued the dissection below it. More coagula were now removed, and the tendinous expansion formed by the vastus internus and adductor longus was then exposed. The external incision being scarcely sufficiently free, was enlarged, when the vena saphena major, or a branch of it, was wounded, and bled pretty smartly, though the bleeding was readily controlled by pressure with the finger. A puncture in the sheath was now apparent, and Mr. Keate was proceeding to slit up the latter on a grooved director, when suddenly the compression at the groin, which had hitherto completely restrained any hæmorrhage from the femoral vessels, proved ineffectual, or slipped from its place, and the wound was in an instant filled with blood, *both arterial and venous*. Mr. K. with great presence of mind, thrust his hands into the wound, whilst an assistant made pressure on the vessels where they lie on the bone, beneath Poupart's ligament, and a tourniquet, at the same time, was quickly slipped upon the limb, and screwed up tight, when the bleeding ceased. Some venous blood, however, probably that locked up in the limb, continued to flow for some seconds more, when it ceased of its own accord. All being thus secure, Mr. Keate completed the opening in the tendinous sheath on a director, discovered the femoral artery, distinctly saw the puncture in its coats, and passed an aneurismal needle, armed with a double ligature, beneath it, carefully avoiding the femoral vein. The needle was removed, the two threads of the ligature separated, and one tied above, the other below, the wound in the vessel. The screw of the tourniquet, after this, was loosened; but the venous hæmorrhage again burst out, and, afraid of trusting to compression alone, in the patient's exhausted state, as well as despairing of tying the vein by itself, in the midst of such effusion of blood, in so deep a situation, Mr. Keate again introduced the aneurismal needle, armed with a double ligature, as before, separated the threads, and tied both artery and vein together, above and below the former ligatures on the artery alone. The bleeding then ceased for good and all —the wound was sponged clean—the lips brought together by four strips of plaister, having an interval of a quarter of an inch between each—light compresses were applied, and, lastly, some turns of a roller. Each ligature embracing the artery *and* vein at the top and bottom of the wound, was distinguished from the two on the artery alone, in the middle, by a knot.

The man, the operation and dressings being finished, was undressed—the limb directed to be rolled in flannel—and some weak wine and water, and afterwards warm tea, administered for drink. He bore the operation with the utmost fortitude, and after its conclusion, the pulse was but little weaker than at its commencement. Mr. Keate displayed throughout that coolness and presence of mind for which he is distinguished in his operations.

It will be observed that there were four ligatures, in all, upon the artery, and only two upon the vein.

*a a* Artery.
*b b* Vein.
*c c* Ligatures on Artery.
*d d* Ligatures on Artery and Vein, distinguished by a knot.

Twenty-five minims of the liquor opii sedativus were immediately given to the patient, and at 10, p.m. when we saw him, he complained of a good deal of pain in the limb, not, however, exclusively following the course of the nervus saphenus. The pulse was something quicker and fuller—the manner tranquil—the foot cool. He was shivering, and had been very chilly ever since the operation.

*Repetatur haust. liq. op. sedativ.*

4th. The countenance is anxious, and the cheek tinged with a slight and hectic flush—the tongue is dry, and of a glazed brightish red at the sides and extremity—the pulse rapid, weak, and expressive of the highest irritability—the great toe rather cool, but the rest of the foot and the limb quite warm. He has suffered since early in the morning from sickness, and complains of much pain on the inside of the thigh. The roller was removed, with the effect of procuring relief to the pain—the limb was laid gently on its outer side—the knee somewhat bent—and some compresses and a turn or two of the roller, wetted with a lotion, composed of three parts of rectified spirits of wine to nine of camphor mixture, lightly re-applied.

℞. *Sod. subcarb. gr.* xx. *Succi limon.* ℥ss. *Liq. op. sedativ.* ♏xx. *Aq. distil.* ℥j. *M. ft. haust. statim sumend. inter effervescendum.*

*Sod. subcarb. gr.* xx. *Succi limon.* ℥ss. *Aq. distil.* ℥j. *4tis horis inter effervescend.*

In the afternoon of the 4th, the irritability being extreme, he was ordered another dose of the liquor opii sedativus, after which he passed a tolerable night, and the sickness subsided. On the 5th, however, he was very ill; the pulse was 150—the tongue even still more extensively glazed and dry—the cheeks suffused with a hectic flush—the expression, as if he was inclined to be light-headed, which, indeed, he said that he was. He complained of no pain in any part, but the thigh was more swelled, and, on changing the straps, about a tea-spoonful of blood came away from the upper part of the wound, but the hæmorrhage, if such it may be called, instantly and spontaneously stopped. Some strips of plaister were lightly re-applied, with a compress or two of lint, and rags wetted in the before-mentioned lotion. He was ordered (he had before taken some gruel) a little, and but a little, light nourishment, with an ounce of senna draught, as the bowels had not yet been opened. In the afternoon, the house-physic was followed by half an ounce of castor oil, and, in the evening, he took a draught, consisting of twenty-two grains of carbonate of ammonia, half an ounce of lemon-juice, forty minims of the liquor opii sedativus, and an ounce of distilled water, in a state of effervescence.

He passed an indifferent night, and on the 6th, was in a very unfavourable state.

The tongue was much the same as before—the pulse 160 and upwards, not small, but yet possessing not the slightest force—the face not so flushed, but more muddy and anxious—the mind wandering—the stomach irritable. He had had three motions—no chilliness or rigor. On the inside of the thigh was observed the commencement of that state which rapidly seized on the limb in the last few hours of existence. It consisted in a dusky and indistinct blush, with pain on pressure, and tenderness, (so it was thought,) in the course of the femoral vein, and in the hypogastrium. The foot was still warm, but he scarcely felt when the toe was touched.

℞. *Ext. hyosciam.* gr. iv.

*Potass. subcarb.* gr. xviij.—*succ. limon.* ℥ss.—*liq. ant. tart* m. xij.—*tinct. hyosciam.* m. xl.—*aq. distill.* ℥j.—*syrup.* ʒiss. ℳ. 4*tis horis.*

That night he was so delirious and violent that he was obliged to be held in his bed, and 3, p. m. of the 7th, when we saw him, he certainly seemed to be dying. There was constant dribbling of blood from the nose, the aspect was sunken and wild, the pulse rapid and quivering, the tongue almost black, the teeth incrusted, the mouth surrounded with herpetic eruptions. He complained of some, but not much pain in the limb, which we did not see, as it was wrapped in flannel, and it really would have been cruel to disturb the patient. Mr. Harrison, however, the house-surgeon, informed us that he saw no distinct appearance of gangrene, though the temperature of the limb was sunk.

The prostration increased, and death took place in the course of that evening.

On looking at the limb next day, Sunday, in the dead-house, it certainly appeared in a state of gangrene. It was mottled in colour from the foot to the hip, and from that to the axilla, the same kind of patches, but fainter in hue, were observed. The belly was as tense and sonorous as a drum, the skin generally of a yellowish tinge. On the inside of the thigh was a large vesication, the wound was very sloughy, and over the internal malleolus and heel was another extensive vesication, filled with dark modena-coloured serum. The dissection of the body did not actually take place till 1, p. m. of the 9th, Mr. Keate deferring it till Monday, in order that the pupils, &c. might be present.

*Sectio Cadaveris.* The wound and the parts in it were in such a sloughy disorganized state, that nothing at all satisfactory could be made out. The two upper ligatures were attached so slightly to the vessels, that they came away during the examination, and no one could venture to pronounce whether they had or had not been thrown off by sloughing. The nervus saphenus passed distinctly to the outside of the vessels, and was perfectly intact. Above the ligatures no coagulum was found in the femoral artery or vein—below, we believe, there were coagula in both. There was not the slightest trace of inflammation in the femoral vessels, but the inner coat of the vein was stained of a deep cherry colour, and that of the artery was also stained in a less degree. The wound was in nearly the centre of a cavity passing deep behind the bone, and containing sloughing cellular membrane and pus, whilst all the muscles and textures in the neighbourhood were more or less disorganized. The cellular tissue passing up between the adductor muscles was likewise in a sloughy state, and on cutting across the sartorious muscle, pus was found to be deposited in its substance, the depôt being unconnected with the matter around. The subcutaneous cellular membrane of the leg was in general not much diseased, but the cutis and cuticle in many parts were dying, and the deeper muscles, on being cut into, were completely rotten and gangrenous.

On opening the abdomen the intestines were found much distended with air, and presented, in many parts, a mottled appearance, arising from staining of the coats with bile, which was present in considerable quantities. The liver was generally pale, and, as it seemed, bloodless, extraordinarily soft in its texture, and marked here and there with spots like ecchymoses or petechiæ. They were, in fact, small extravasations of blood in the hepatic parenchyma. There was an unusual quantity of water in the pericardium, and some in each side of the chest; but the lungs and the heart were sound, and the pleuræ presented no marks of inflammation. The blood throughout the body presented a decided tendency to fluidity.

The head was not examined.

There cannot be a doubt that wound of the femoral artery is always to be regarded as a dangerous, we do not by any means say fatal, accident, not merely in its primary but secondary effects. The ligature of the main arterial trunk of the lower extremity for a wound of it, is cæteris paribus, much more to be dreaded, quoad the circulation of the limb below, than ligature of the same vessel in the same situation for aneurism. If both artery and vein be wounded, and especially if the wound be gun-shot, the danger is proportionately increased, and there are cases enough on record to prove, that the chances of gangrene of the limb are very considerable indeed. Mr. Guthrie, in his work upon gun-shot wounds, a work which every surgeon should possess, has made the following observations on this subject.

"I by no means intend to assert, that the anastomosing branches of arteries are not equal to carry on the circulation in the extremities in every instance, where the main artery has been wounded, for I know the contrary; indeed, in the upper extremity it will almost invariably be effected; but in the lower, where there has been no previous disease, and the femoral or popliteal artery be divided by a musket-ball, the anastomosing branches cannot always carry on the circulation, and sphacelus will affect the toes. I think I have seen it cease at a part of the ball of the great toe, in an unsuccessful case of femoral artery tied after a gun-shot wound; and I have seen it in other cases destroy the patient. If the vein accompanying the artery be injured, I believe mortification of the extremity to be inevitable; and in gun-shot wounds there is frequently more or less injury of the vein, as well as of the artery."

Mr. Hodgson remarks, that "it must be acknowledged, however, that the mortification of the limb is a more frequent occurrence after the ligature of a wounded artery than when an artery is tied for the cure of an aneurism." Mr. Samuel Cooper has been at some pains to prove, or to attempt to prove, that the fears of gangrene are unfounded, and the ligature of the principal artery of a limb as safe as in the case of aneurism. Mr. Cooper, however, might have saved himself a vast deal of valuable argumentation, for all who will peruse the cases recorded in the fasti of surgery, will find, no matter what dicta exist to the contrary, that mortification *is* a too frequent sequence of such an injury and such an operation.

Of course we do not intend to assert that mortification of the limb must necessarily follow the sudden obstruction by ligature of the main arterial trunk, for we know full well that such is not the case, in the upper extremity especially. What we mean to say is this, that, consideratis considerandis, the chances of such occurrence in such particular circumstances are considerable, and when the artery and vein of the lower extremity are wounded, we do believe that Mr. Guthrie is right in his principle, and that death of the limb will, in all probability, follow. Holding these opinions, we were little surprised at the termination of the present case, which goes as far as one case can go to support and to confirm them. We are disposed, however, to think, that something depends on the kind of injury, as well as on the mere obstruction of the vessels. It appears to us that a wound from gun-shot which lacerates and bruises the neighbouring parts, as well as implicates the artery and vein, is even more certain to produce mortification than a clean, small puncture or stab in the vessels. In the latter case you have only the deficient circulation to deal with, in the former, the inflammation or sloughing in addition, which follow a lacerated wound. In conclusion, then, we would remark on the subject of mortification, that it frequently takes place after wound and ligature of the femoral artery only, and almost always when the vein and artery both have been transfixed. We have said almost always, for a case has been recorded by the Editor of this Journal of wound of these vessels in the sheath of the triceps by a knife, which occurred in one of His Majesty's ships at Spithead, when the artery was tied above the wound, and gangrene did not ensue.* It is possible that other similar cases have occurred, for every one knows the uncer-

* As the case is short, and one of great rarity, we are tempted to extract it entire from the ninth volume of the New Medical and Physical Journal, in which it was detailed.

tainties of surgery as well as of physic, but whether they have or have not, there are instances enough on the other side of the question to prove to every unprejudiced man, that these must be viewed as the exceptions, the supervention of mortification as the rule.

As for the operation performed in the present instance, it is generally, we believe, conceded amongst surgeons, that wounds of large arteries are not to be treated like aneurismal affections of the same trunks by a ligature at a greater or less distance from the spot, but the vessel is to be tied above and below. We believe that this is the general opinion, but we know that it is not the universal one, and that some whom we highly respect for their station and acquirements are quite of a different way of thinking on the subject. This, however, is a ques-

---

" *Wound of the Femoral Artery, Femoral Vein and Crural Nerve.*—(Communicated by Dr. Johnson.)—A young man belonging to a transport at Spithead, received a wound in the thigh, a few days ago, by a large knife, which entering the limb crosswise, divided the femoral artery, vein, and, it was supposed, the nerve, at the point where the femoral artery penetrates the triceps in its passage to the ham. There was no medical assistance in the vessel, and a tremendous hæmorrhage reduced the unfortunate sufferer, in a few minutes, to the verge of death. Deliquium animi, however, suppressed for a time the effusion of blood, and a young assistant Surgeon from a gun-brig, the Teazer, (Mr. Lawrence M'Kay, a promising young gentleman) that lay near the transport, arrived just soon enough to apply a tourniquet, as the reviving spark was giving rise to a second hæmorrhage which would have instantaneously proved fatal !—In this state he was conveyed to the Royal Hospital at Haslar, where the steps that were to be taken required a discrimination that might not be considered necessary or difficult by a great number of Surgeons, though the case involved very nice points of practice, and was calculated to give birth to much bad surgery, among unreflecting operators.

" From the site of the wound, and the dislocation (if the term be allowed) of relative situation in the parts wounded, it was found to be impracticable, or at least extremely difficult, to secure the vessels at the point where they were severed ; and the tourniquet was placed on the spot where the artery is usually taken up in the operation for popliteal aneurism. The young man was scarcely alive, from the profuse loss of blood ; and the great object was now, to secure the vessels without losing a single drop, if possible, of the vital fluid.

" If another tourniquet were applied high up, while the present one was slacked off, and the artery secured in the common place, all the blood between the two tourniquets must be lost ; and a few ounces might now decide the fate of the patient. If the artery were pressed as it passes over the os pubis, till the operation were performed, it would be still worse, for the posterior iliac would supply blood to the wounded femoral vein, and occasion much hæmorrhage. What then was to be done?—Another tourniquet was applied with a proper pad and compress, *on* and somewhat *above* the wound ; so as to compress that end of the artery which poured the blood from above, and that end of the vein which poured the blood from below. The original tourniquet was then taken off, the artery laid bare, and tied with a single ligature. The tourniquet on the wound was then removed, leaving just compression enough, by bandage, to prevent effusion from the femoral vein, but not so much as to obstruct the circulation of the venous blood through the *other* channels of communication with the body, nor the arterial through the branches of the profunda, &c. &c. in its way to nourish the leg. A very slight oozing from the wound took place the first night, since which, every thing went on well, and he is now recovered.

" We thus see that the above Case was an extremely interesting one, and capable of exciting many useful reflections. It may be remarked, that this is the second instance, within these few weeks, where a single ligature was applied to the trunk of the femoral artery with success.

" Dr. Smith of Haslar, was the operating Surgeon on this last occasion."

J. J.

tion which cannot be decided by the experience, however great, or the sentiments, however entitled to our regard, of any single individual, and we do conceive that the operation for aneurism, when applied to wounded arteries, has been found in the practice of the many and in the long run, inappropriate and perilous. All our best writers on surgery condemn it, and facts, established facts, which are worth whole bushels of argument, condemn it also.

On this account we fancy that Mr. Keate's having tied the artery above and below the puncture in its coats, will not be objected to by the majority of surgeons. Probably, however, this may not be the case with the ligature on the vein. It may, perhaps, be said, that compression would have stopped the hæmorrhage from *that*, and so in fact it might, for any thing we know, or that any body else can know to the contrary. This, however, we will say, that we saw the operation, we witnessed the bleeding, and sorry indeed we should have been to have trusted that patient, exhausted as he was, without a ligature on his femoral vein, or to have irritated and fretted such a wound by continued compression. Were the accident to occur to ourselves tomorrow, and the vein to bleed as it bled that night, we would certainly entreat the operator to place a ligature upon it. Such a proceeding, if it have an injurious effect at all, must have it by exciting inflammation in the tube—by producing phlebitis. In the present case, no such phlebitis ensued, and the cases we have witnessed lead us to conclude that the deep-seated veins are not so liable to inflame as the cutaneous, either after injuries or operations. The application of the ligature, which on the saphena would be fatal, may be used with comparative impunity to the femoral, deep in the thigh. Every one must have noticed how frequently veins are secured on the surface of stumps, and yet how rarely this is followed by any bad effects.

The double ligature on the vein, in this instance, was perhaps not absolutely necessary; but if mischief was to follow the tying of the vein, it would equally result from one thread as from two; and besides, in an operation of this kind, things are often done on the spur of the moment, which might not be attempted if the surgeon had leisure to arrange every step with precision beforehand. It is a very different thing to criticise at one's ease, and perform an operation in the midst of blood, and hurry, and confusion.

The symptoms which the patient presented, up to the last day, were those of the most marked irritation which we ever remember to have witnessed. These are the cases which perplex in practice; where we fear to stimulate and dare not deplete. Towards the last, the irritative fever gave way to that of a typhoid stamp, evinced by black tongue, delirium, and hæmorrhage from the nose. These phenomena, we suppose, were symptomatic of the gangrene invading the limb; but certainly any one looking at the man would have thought that he was dying of a putrid fever.

The case altogether is one of such interest, that we trust the length at which we have related it will not be considered by our readers an unpardonable offence.

---

## Figures and Tables

**Figure f1:**